# Supplementary material for: Insights into Dermal Permeation of Skin Oil Oxidation Products from Enhanced Sampling Molecular Dynamics Simulation
Source: J Phys Chem B. 2025 Feb 4;129(6):1784–94. doi: 10.1021/acs.jpcb.4c08090 (PMC11831647; doi:10.1021/acs.jpcb.4c08090)
Supplement: Supplementary file 1 — jp4c08090_si_001.pdf [file jp4c08090_si_001.pdf]

Supporting Information: Insights into Dermal Permeation of Skin Oil Oxidation Products from Enhanced Sampling Molecular Dynamics Simulation

Rinto Thomas[†,¶], Praveen Ranganath Prabhakar[‡,¶], Douglas J. Tobias[‡,*], and Michael von Domaros[†,*]

[†]Fachbereich Chemie, Philipps-Universität Marburg, 35032 Marburg, Germany
[‡]Department of Chemistry, University of California, Irvine, Irvine, California, 92697 United States
[¶]R.T. and P.R.P. contributed equally to this work.

E-mail: dtobias@uci.edu; mvondomaros@uni-marburg.de

1 Supplementary Methodological Details

1.1 Details on the POPC System

We simulated two POPC systems with slight differences in size, containing 72 and 128 lipids, respectively. Initial structures for both were obtained from the CHARMM-GUI Web site.[1] Simulations were conducted at two close temperatures, 303.15 K and 305.15 K, with specific size-temperature combinations detailed in Table S1. These variations allow direct comparisons with previous studies.[2,3] However, for our main argument—that simulation times are significantly longer in SC vs. POPC—these minor differences in system size and temperature are negligible.

Table S1: System size and temperature combinations used in the POPC simulations.

	US		WTM	
System	No. of Lipids	Temperature	No. of lipids	Temperature
POPC/Water	72	305.15 K	72	303.15 K
POPC/Acetone	72	305.15 K	128	303.15 K

1.2 Error Analysis

Standard errors for the FE profiles obtained from US were estimated as described by Hummer et al.,[4]

$$\mathsf{var}[F(z_n)] \approx \left(\frac{K}{2}\Delta z\right)^2 \cdot \sum_{i=0}^{n} \mathsf{var}(\bar{z}_i), \tag{S1}$$

where K and Δz have already been defined in the main text and correspond to the harmonic force constant and the spacing between two adjacent umbrella potentials, respectively. Furthermore, $\mathsf{var}(\bar{z}_n)$ denotes the squared standard error of the mean of the collective variable z evaluated for the nth window. These were evaluated by applying the automated blocking method described by Jonsson.[5]

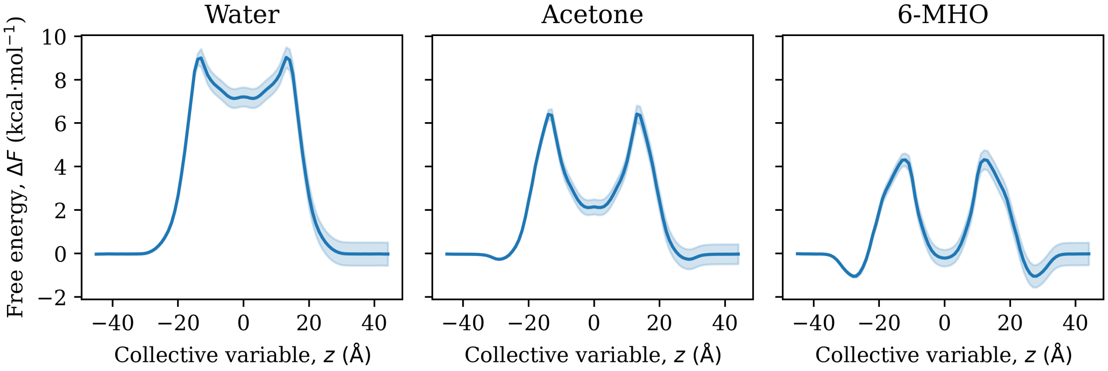

Figure S1: Error propagation through multiple windows in US simulations.

On the left-hand side of the equation, $\mathsf{var}[F(z_n)]$ denotes the squared standard error of the free energy evaluated at z_n. Note that errors accumulate over the windows, that is, they are asymmetric by construction (Figure S1). This is reasonable since the first window, $n = 0$, is chosen as the reference point for the FE profile and cannot have an error by definition. To obtain a symmetrized version of the error bars, we carried out the same procedure, but treated both halves of the membrane as independent samples. To be precise, we evaluated Eq. S1, both for $n = 0 \ldots n_{\mathsf{max}}/2$ and for $n = n_{\mathsf{max}} \ldots n_{\mathsf{max}}/2$, where $n_{\mathsf{max}} = 90$ in our simulations. We then averaged over pairs of symmetry-equivalent FE values, using the laws for error propagation of uncorrelated random variables,

$$F^{\mathsf{sym}}(z_n) = \frac{1}{2}\left[F\left(z_n\right) + F\left(z_{n_{\mathsf{max}}-n}\right)\right] , \tag{S2}$$

$$\mathsf{var}[F^{\mathsf{sym}}(z_n)] = \frac{1}{4}\left\{\mathsf{var}[F(z_n)] + \mathsf{var}[F(z_{n_{\mathsf{max}}-n})]\right\} . \tag{S3}$$

To obtain the final FE profiles that span the entire CV space, we mirrored the symmetrized free energies and errors with respect to the center of symmetry ($z = 0$).

We adopted a similar strategy for the WTM simulations. Here, snapshots of the FE profiles were saved every 2 ns. Each of these FE profiles was bifurcated with respect to the center of symmetry and referenced so that the FE in the center of the aqueous region is 0. Finally, Jonsson's automated blocking method was applied to each half FE profile and averages were computed as described above.

1.3 Number of Contacts

The number of contacts N between a solute (s) and the lipids (l) was calculated using

$$N = \sum_l \frac{1 - \left(|\mathbf{r}_s - \mathbf{r}_l| / d_c\right)^n}{1 - \left(|\mathbf{r}_s - \mathbf{r}_l| / d_c\right)^m} . \tag{S4}$$

Here, $\mathbf{r}_s$ refers to the position of the oxygen atoms of the solutes (for water: hydroxyl, for acetone, 6-MHO: carbonyl), $\mathbf{r}_l$ denotes the position of the hydroxyl oxygen atoms of the lipids, $d_c = 4$ Å is the cutoff distance, and $n = 6$ and $m = 12$ are common parameter choices that ensure that the function decays to zero quickly for separations larger than d_c. The sum runs over all lipids. For ceramides, which have two hydroxyl groups, we present averages.

2 Supplementary Results

2.1 Density Profile

We computed the mass density profile ρ in bins of size $\Delta z = 0.1$ Å along the collective variable for the SC bilayer (Figure S2). The profile is perfectly symmetric, despite the lipid flipping described in the main text. It permits an easy identification of the aqueous phase, the lipid headgroup region, and the tails.

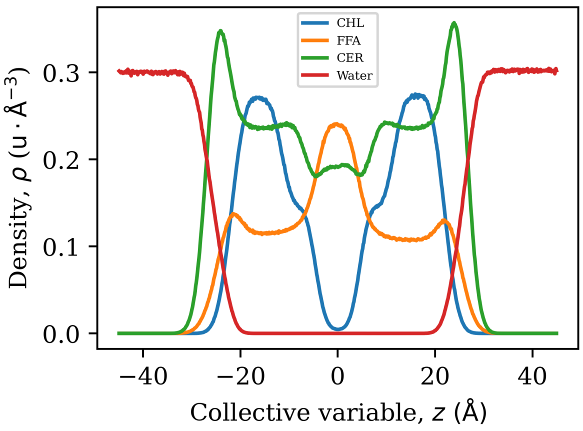

Figure S2: Density profiles of the FFA, CHL, CER and water in the SC system.

2.2 Free Energy Profiles

Figures S3 and Figure S4 are the equivalents of Figures 3 and 4 for POPC, showing the convergence toward symmetry of the FE profiles. In Figure S5, we give the final force-symmetrized PMFs for the SC system in its raw version, without using smoothing. In Figure S6 we give the final force-symmetrized PMFs for the POPC system.

For acetone, we find qualitative differences to similar results reported in the literature, which are likely due to differences in the force fields used to model the interactions in the system (Gromos / OPLS / SPC vs Charmm / CGenFF / TIP3P).[2]. However, we note that our results are qualitatively in agreement with the octanol–water partition coefficient reported by Cumming et al.[6] ($\log K_{\mathrm{OW}} = -0.22$), indicating that it is thermodynamically unfavorable for acetone to partition from water to a lipophilic region.

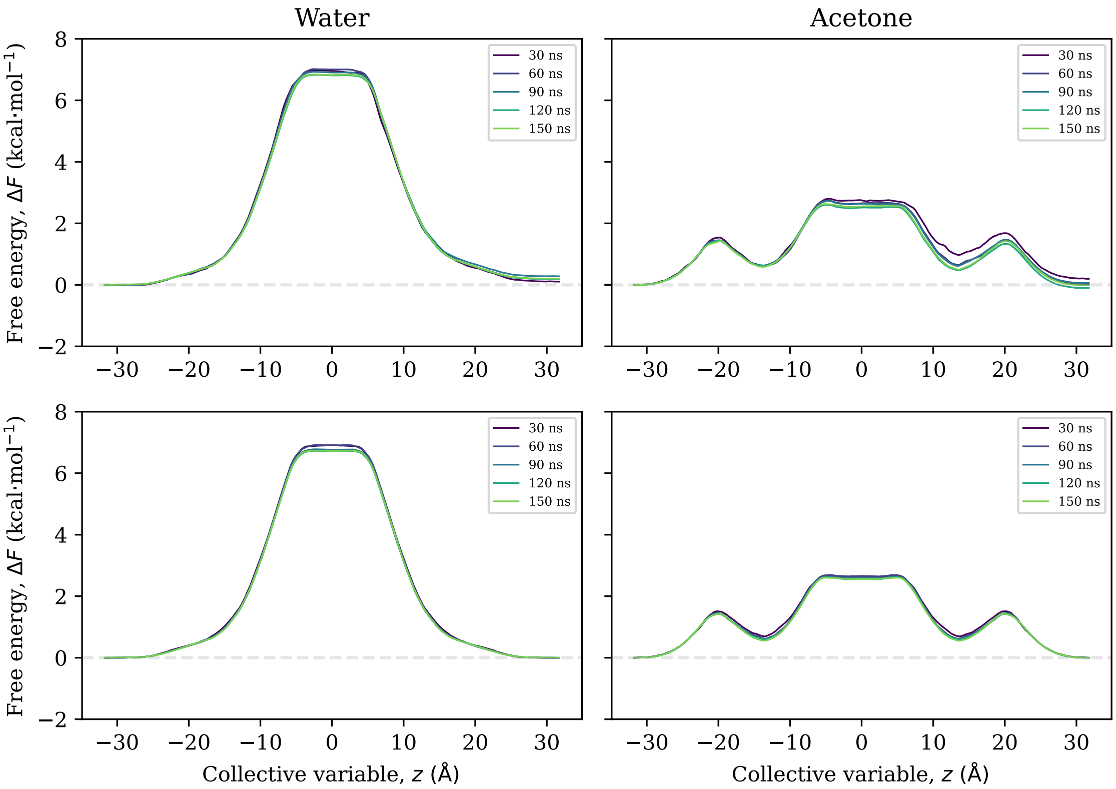

Figure S3: Top row: Convergence of POPC FE profiles toward symmetry for US. Bottom row: Convergence of the corresponding force-symmetrized FE profiles toward their final values. All simulation times are per window. The total simulation times, which we report in Table 1, are in the µs region.

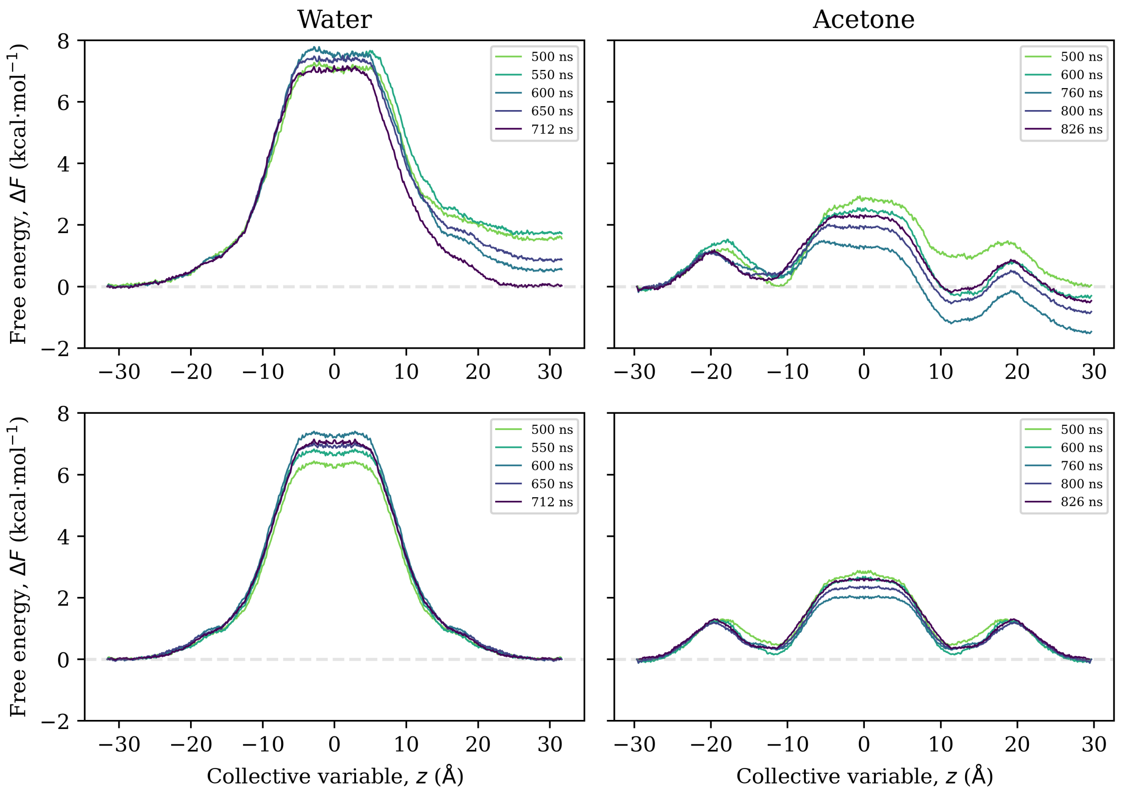

Figure S4: Top row: Convergence of POPC FE profiles toward symmetry for WTM. Bottom row: Convergence of the corresponding force-symmetrized FE profiles toward their final values.

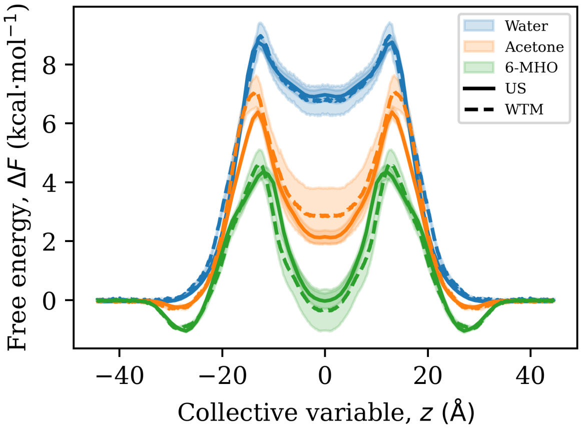

Figure S5: Final, force-symmetrized FE profiles for all investigated solutes in the SC model. Unsmoothened version of Figure 5 shown in the main text.

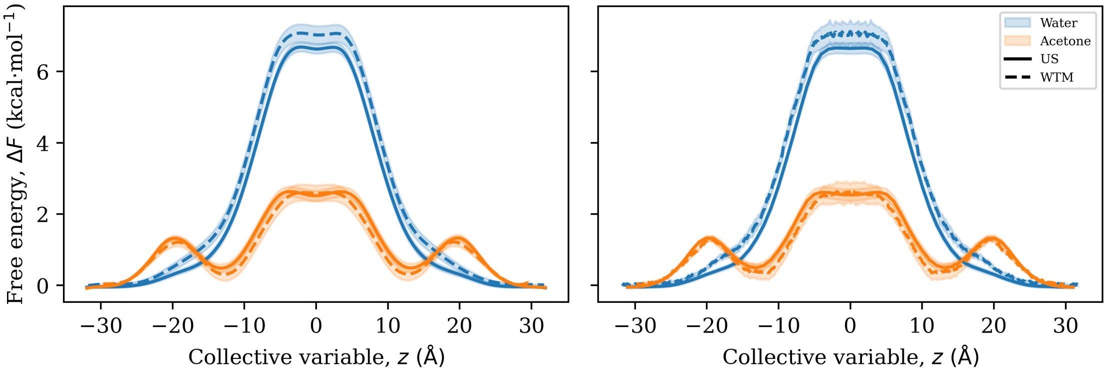

Figure S6: Final, force-symmetrized FE profiles for all investigated solutes in the POPC model with smoothened version in the left panel and the unsmoothened version in the right panel

2.3 Order Parameters

To understand the dip in the order parameters observed in the oleoyl chain of POPC and the sphingosine chain of the ceramide lipids, we present here distributions of the cosine of the angle formed by methylene bonds and the bilayer normal. Averaging over the second Legendre polynomial of this cosine gives the order parameter,

$$S_{\mathrm{CH}} = \frac{\langle 3\cos^2\theta - 1\rangle}{2}. \tag{S5}$$

To construct the plots shown in Figures S7 and S8, the same trajectory was analyzed as used to calculate the order parameters (Figure 6 in the main text). For better clarity, only the lipids in the upper leaflet of the membrane were considered. The data points were grouped into 100 bins of equal width, and the final distribution was normalized to provide the probability density.

While the methylene bonds adopt a range of orientations in both systems, the distributions are not uniform but are centered at $\cos(\theta) \approx \pm 0.57$, which corresponds to the "magic" angle, for which $S_{\mathrm{CH}} = 0$. These findings explain why the order parameter nearly vanishes for double-bonded carbon atoms in POPC ($|S_{\mathrm{CH}}| \approx 0.05$) and why it has a relatively low value in sphingosine ($|S_{\mathrm{CH}}| \approx 0.13$).

We note that similar findings have been reported by other authors.[7–9]

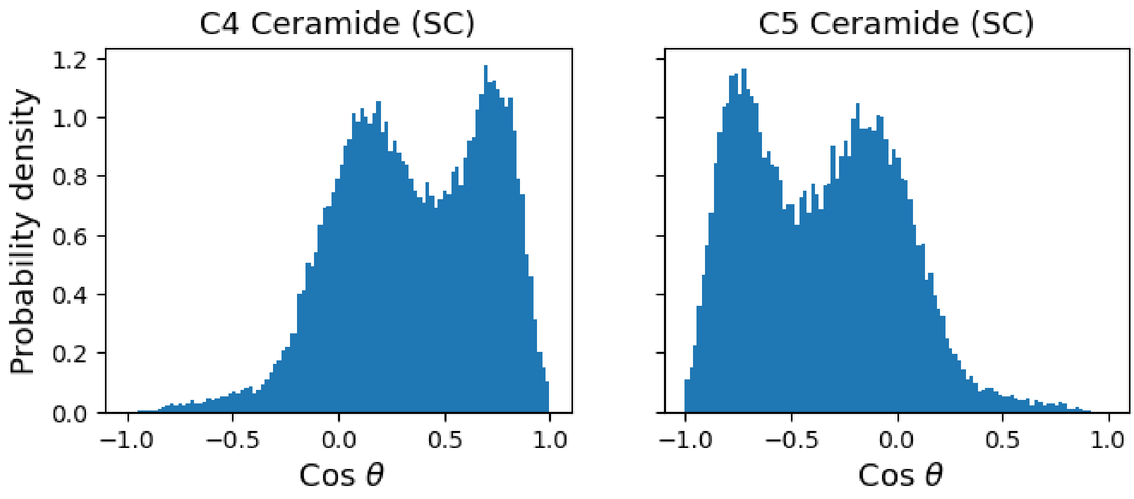

Figure S7: Distribution of the cosine of the angle formed by the sphingosine methylene bonds (located at C4 and C5) and the bilayer normal.

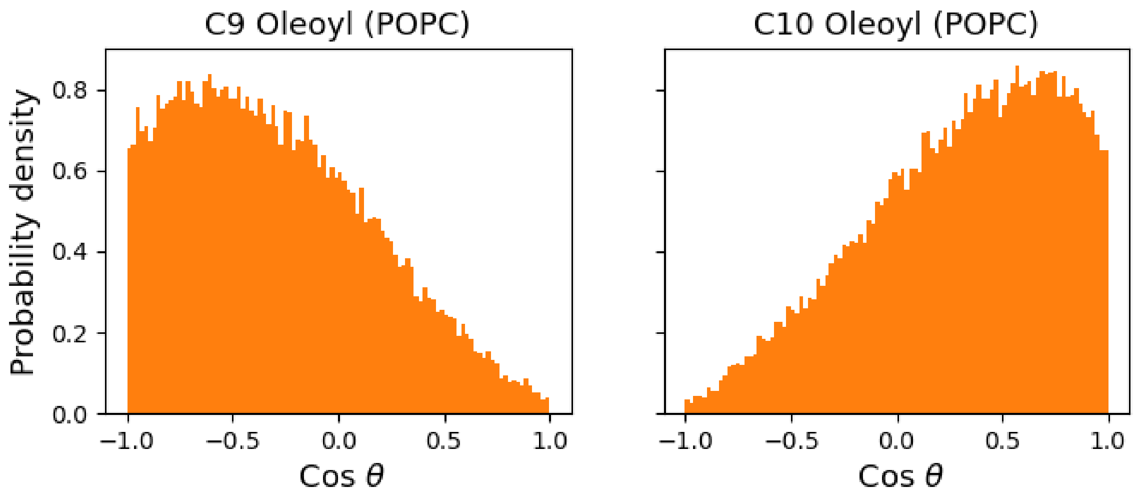

Figure S8: Distribution of the cosine of the angle formed by the oleoyl methylene bonds (located at C9 and C10) and the bilayer normal.

2.4 Number of Contacts

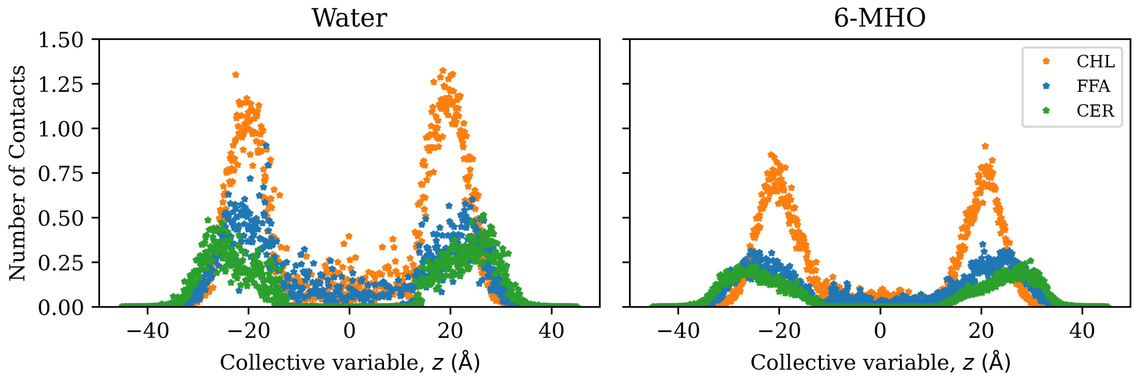

Figure S9: Average coordination number between SC lipids and water (left panel) as well as 6-MHO (right panel), stratified by the position along the membrane normal.

2.5 Flipping Events and Mechanisms

Table S2: Summary of all flipping events observed in the WTM and unbiased simulations. The mechanisms a-d have are described as follows; (a) solute assisted, (b) solute and solvent assisted (c) solvent assisted and (d) unassisted.

System	Residue	Start (ns)	Stop (ns)	Mechanism
Water in SC	CHL	49	367	d
Water in SC	CHL	843	1113	b
Water in SC	FFA	122	125	d
Water in SC	FFA	371	393	b
Acetone in SC	CHL	3177	3419	d
Acetone in SC	FFA	45	48	a
Acetone in SC	FFA	100	115	a
Acetone in SC	FFA	646	704	a
Acetone in SC	FFA	928	1132	a
Acetone in SC	FFA	1717	1998	a
6-MHO in SC	CHL	61	119	a
6-MHO in SC	CHL	197	502	a
6-MHO in SC	CHL	836	1035	b
6-MHO in SC	CHL	1097	1172	a
6-MHO in SC	CHL	5967	5978	a
6-MHO in SC	FFA	419	565	b
6-MHO in SC	FFA	4327	4382	c
6-MHO in SC	FFA	6517	6657	d
Unbiased Neat SC	CHL	33	199	d

References

[1] Jo, S.; Kim, T.; Iyer, V. G.; Im, W. CHARMM-GUI: A Web-Based Graphical User Interface for CHARMM. *J. Comput. Chem.* **2008**, *29*, 1859–1865.

[2] Posokhov, Y. O.; Kyrychenko, A. Effect of Acetone Accumulation on Structure and Dynamics of Lipid Membranes Studied by Molecular Dynamics Simulations. *Comput. Biol. Chem.* **2013**, *46*, 23–31.

[3] Krämer, A.; Ghysels, A.; Wang, E.; Venable, R. M.; Klauda, J. B.; Brooks, B. R.; Pastor, R. W. Membrane Permeability of Small Molecules from Unbiased Molecular Dynamics Simulations. *J. Chem. Phys.* **2020**, *153*, 124107.

[4] Zhu, F.; Hummer, G. Convergence and Error Estimation in Free Energy Calculations Using the Weighted Histogram Analysis Method. *J. Comput. Chem.* **2012**, *33*, 453–465.

[5] Jonsson, M. Standard Error Estimation by an Automated Blocking Method. *Phys. Rev. E* **2018**, *98*, 043304.

[6] Cumming, H.; Rücker, C. Octanol–Water Partition Coefficient Measurement by a Simple ^{1}H NMR Method. *ACS Omega* **2017**, *2*, 6244–6249.

[7] Seelig, J.; Waespe-Sarcevic, N. Molecular Order in Cis and Trans Unsaturated Phospholipid Bilayers. *Biochem.* **1978**, *17*, 3310–3315.

[8] Niemelä, P.; Hyvönen, M. T.; Vattulainen, I. Structure and Dynamics of Sphingomyelin Bilayer: Insight Gained through Systematic Comparison to Phosphatidylcholine. *Biophys. J.* **2004**, *87*, 2976–2989.

[9] Merz, K. M. Molecular Dynamics Simulations of Lipid Bilayers. *Curr. Opin. Struct. Biol.* **1997**, *7*, 511–517.
